# Modelling vitamin D food fortification among Aboriginal and Torres Strait Islander peoples in Australia

**DOI:** 10.1038/s41430-026-01737-y

**Published:** 2026-04-09

**Authors:** Belinda Neo, Noel Nannup, Dale Tilbrook, Eleanor Dunlop, John Jacky, Carol Michie, Cindy Prior, Brad Farrant, Carrington C. J. Shepherd, Lucinda J. Black

**Affiliations:** 1 Curtin Medical School, Curtin University, Bentley, WA Australia; 2 The Kids Research Institute Australia, Nedlands, WA Australia; 3 Maalinup Aboriginal Gallery, Caversham, WA Australia; 4 Curtin School of Population Health, Curtin University, Bentley, WA Australia; 5 Institute for Physical Activity and Nutrition (IPAN), School of Exercise and Nutrition Sciences, Deakin University, Geelong, VIC Australia; 6 Ngangk Yira Institute, Murdoch University, Murdoch, WA Australia

**Keywords:** Nutrition, Public health

## Abstract

**Background/Objective:**

Low vitamin D intake and prevalence of serum 25-hydroxyvitamin D concentration <50 nmol/L among Aboriginal and Torres Strait Islander peoples highlight a need for public health strategies to improve vitamin D status. Since few foods contain naturally occurring vitamin D, food fortification could be a suitable strategy. We aimed to model vitamin D food fortification scenarios among Aboriginal and Torres Strait Islander peoples.

**Methods:**

We used nationally representative food consumption data (*n* = 4109, aged ≥2 years) and vitamin D food composition data to model four scenarios. Scenario 1 included the maximum vitamin D concentrations permitted for fortification in Australia: i) dairy products and alternatives, ii) butter/margarine/edible oil spreads, iii) formulated beverages, and iv) selected ready-to-eat breakfast cereals. Scenarios 2a-c included some vitamin D concentrations higher than permitted in Australia. Scenario 2a: i) dairy products and alternatives, ii) butter/margarine/edible oil spreads, iii) formulated beverages. Scenario 2b: as per Scenario 2a plus selected ready-to-eat breakfast cereals. Scenario 2c: as per Scenario 2b plus bread (not permitted for vitamin D fortification in Australia).

**Results:**

Vitamin D fortification could potentially increase median vitamin D intake by ~3–6 µg/day. Scenario 2c had the highest potential increase in median vitamin D intake at ~8 μg/day. Across all modelled scenarios, none of the participants had vitamin D intake above the Australian upper level of intake of 80 μg/day.

**Conclusion:**

Vitamin D fortification could increase vitamin D intake among Aboriginal and Torres Strait Islander peoples, even considering the restrictions on the foods permitted for fortification in Australia.

## Introduction

Aboriginal and Torres Strait Islander peoples are the First Nations peoples of Australia. Before colonisation, around 250 years ago, Aboriginal and Torres Strait Islander peoples largely maintained disease-free lives by following their traditional physically active lifestyle and nutrient-rich diet [1]. Colonisation disrupted those food systems; government rations were first imposed, followed by a Western dietary pattern characterised by energy-dense foods high in sugar, salt and fat, resulting in significant changes to their dietary intake [1, 2]. The pervasive impact of colonisation has led to the loss of traditional food practices, food insecurity, and health inequities for Aboriginal and Torres Strait Islander peoples [2].

Low vitamin D status is a prevalent public health issue in Australia and may be associated with adverse musculoskeletal health and other non-communicable diseases [3, 4]. Based on nationally representative data from 2022–2024, the prevalence of serum 25-hydroxyvitamin D (25(OH)D) concentration <50 nmol/L among the general Australian population was ~29% among adolescents aged 12–17 years, and ~21% among adults aged ≥18 years [5]. Aboriginal and Torres Strait Islander peoples make up ~4% of the Australian population [6], and the prevalence of serum 25(OH)D concentration <50 nmol/L in 2022–2024 was ~23% among adolescents aged 12–17 years and even higher among adults aged ≥18 years, at ~27% [7]. We previously reported a median vitamin D intake of 2 μg/day for Aboriginal and Torres Strait Islander peoples aged ≥2 years [8]. For context, the estimated average requirement for vitamin D intake recommended by the National Academy of Medicine is 10 μg/day for individuals aged ≥1 year, assuming minimal sunlight exposure [9]. Hence, exploring strategies for improving vitamin D intake and status among Aboriginal and Torres Strait Islander peoples is warranted.

Fortifying widely consumed foods could improve vitamin D intake without requiring major changes to cultural food practices [10, 11]. In Australia, vitamin D fortification is mandated only for edible oil spreads, such as margarine, at a minimum concentration of 5.5 µg/100 g [12]. Milk, yoghurt, and other products may be voluntarily fortified [13], but this practice does not occur routinely. Dietary modelling has been used to assess potential vitamin D food fortification strategies for several countries, including the United Kingdom [14], the Republic of Ireland [15], Belgium [16], Finland [17], and Australia [18, 19]. Those studies showed that fortifying only one or a few staple food products may limit population reach [14, 18, 19]; expanding fortification across a broader range of food products would be more effective [15–17, 20]. We aimed to model vitamin D food fortification scenarios using a range of staple foods to explore potential strategies for improving vitamin D intake among Aboriginal and Torres Strait Islander peoples in Australia.

## Methods

### Study population

The 2012–2013 National Aboriginal and Torres Strait Islander Nutrition and Physical Activity Survey (NATSINPAS) provided the most recent food consumption data for Aboriginal and Torres Strait Islander peoples. A detailed description of the methods is available elsewhere [21, 22]. In brief, the NATSINPAS was conducted between August 2012 and July 2013 among Aboriginal and Torres Strait Islander peoples living in remote and non-remote areas. The Australian Bureau of Statistics (ABS) determined the number of participants for remote and non-remote areas using the 2011 Census counts where available [22]. For areas not covered by the 2011 Census counts and for remote discrete communities in New South Wales, Queensland, South Australia, Western Australia, and the Northern Territory, the number of participants was determined using the 2006 Census counts [22]. Participants were first screened to confirm that they were of Aboriginal and Torres Strait Islander descent, before a suitable time was arranged for an interview. In each dwelling, one adult household member (aged ≥18 years) and, where applicable, one child (aged 2–17 years) was selected for an interview. For children aged 2–17 years, the interview was conducted with a representative adult household member acting as a proxy, with children aged 6–14 years encouraged to participate. Adolescents aged 15–17 years could be interviewed directly with parental or guardian consent. The first interview was conducted face-to-face (*n* = 4109), and a second telephone interview (*n* = 771) was conducted eight days after the first interview for those living in non-remote areas. There were 4088 participants (99.5%) in the first interview, and data for the remaining 21 participants (0.5%) were imputed by the ABS.

### Food consumption data

A 24-hour food recall was used to collect dietary intake data from participants aged ≥2 years over the previous 24 hours. Detailed methods are reported elsewhere [21, 22]. In brief, the Automated Multiple-Pass Method was used to collect detailed information about dietary intake through systematic question prompts. The Automated Multiple-Pass Method was developed by the United States Department of Agriculture and adapted for use in Australia by the ABS in collaboration with Food Standards Australia New Zealand. The Australian Health Survey food model booklet and bush tucker prompt cards were used to aid participants in describing the quantities of food consumed [22]. Food consumption data collected were coded using the unique 8-digit food code assigned to each food entry in the 2011–2013 AUStralian Food and NUTrient (AUSNUT) database, which contains nutrient composition data for 5740 foods [23].

### Food vehicles for fortification

Foods permitted for vitamin D fortification in Australia, according to the Australia New Zealand Food Standards Code [13], were included in our models: dairy products and alternatives, butter/margarine/edible oil spreads, formulated beverages (ready-to-drink, non-carbonated flavoured water-based beverage that has added vitamins and/or minerals, e.g., bottled water with added sugar, vitamins and minerals [24]), and selected ready-to-eat breakfast cereals (RTEBC). Although bread is not currently permitted for vitamin D fortification in Australia, it is commonly consumed by Aboriginal and Torres Strait Islander peoples [25] and has been identified as an effective vehicle for fortification in Australia [26]. Bread was, therefore, included in our modelling as a potential candidate for vitamin D fortification. The proportion of Aboriginal and Torres Strait Islander peoples who reported consuming each included food group is presented in Supplementary Table 1.

RTEBC are only permitted for vitamin D fortification if they meet the Nutrient Profiling Scoring Criterion (NPSC) [27]. The NPSC provides a score calculated based on energy, saturated fat, sodium, sugar, the percentage of fruit, vegetables, nuts, legumes, coconut, spices, herbs, fungi, algae and seeds, and, for some foods, protein and dietary fibre [28]. We used the nutrient information based on 100 g of each RTEBC in the 2011–2013 AUSNUT Food Recipe File to determine whether they were NPSC-compliant. Approximately 73% of the RTEBC met the NPSC criteria and were included in our modelling. According to the Australia New Zealand Food Standards Code, the maximum claim for vitamin D concentration is 2.5 μg per normal serving for RTEBC [13]. We considered a normal serving to be 30 g, the recommended standard serving for wheat cereal flakes in the Australian Dietary Guidelines [29].

### Food fortification scenarios

We modelled four food fortification scenarios; the vitamin D concentration used for each scenario is provided in Table 1. The modelling for Scenario 1 included the maximum vitamin D concentrations permitted for fortification in Australia according to the Australia New Zealand Food Standards Code [13]: i) fluid milk and alternatives (0.8 μg/100 g), ii) butter, margarine, and edible oil spreads (16 μg/100 g), iii) dried milk (1.5 μg/100 g), iv) cheese and cheese products and alternatives (6.4 μg/100 g), v) yoghurt and alternatives (1.07 μg/100 g), vi) dairy desserts (1.07 μg/100 g), vii) formulated beverages (0.42 μg/100 g), and viii) NPSC-compliant RTEBC (8.33 μg/100 g). The modelling for Scenarios 2a–c included some vitamin D concentrations higher than permitted in Australia; Scenario 2c included bread, which is not currently permitted for vitamin D fortification in Australia. Scenario 2a: i) fluid milk and alternatives, ii) butter, margarine, and edible oil spreads, iii) dried milk, iv) cheese and cheese products and alternatives, v) yoghurt and alternatives, vi) dairy desserts, and vii) formulated beverages. Scenario 2b: as per Scenario 2a plus NPSC-compliant RTEBC. Scenario 2c: as per Scenario 2b plus bread.

**Table 1:** Food fortification scenarios with the vitamin D concentration for each food.

Scenario	Foods	Vitamin D concentration (µg/100 g)
1	*Scenario 1 includes foods and maximum vitamin D concentrations that are permitted for vitamin D fortification in Australia*.	
	Fluid milk and alternatives derived from legumes	0.8
	Butter, margarine, and edible oil spreads	16
	Dried milk	1.5
	Cheese and cheese products and alternatives derived from legumes	6.4
	Yoghurts (with or without other foods) and alternatives derived from legumes	1.07
	Dairy desserts	1.07
	Beverages containing no less than 0.3% m/m protein derived from cereals, nuts, seeds, or a combination of those ingredients	0.8
	Formulated beverages (e.g., bottled water with added vitamins and minerals)	0.42
	NPSC-compliant RTEBC	8.33
2	*Scenarios 2a-c include some vitamin D concentrations higher than those permitted in Australia. Scenario 2c includes bread, which is not permitted for fortification in Australia*.	
(a)	Fluid milk and alternatives derived from legumes	1
	Butter, margarine, and edible oil spreads	20
	Dried milk	1.5
	Cheese and cheese products and alternatives derived from legumes	6.4
	Yoghurts (with or without other foods) and alternatives derived from legumes	1.07
	Dairy desserts	1.07
	Beverages containing no less than 0.3% m/m protein derived from cereals, nuts, seeds, or a combination of those ingredients	1
	Formulated beverages	0.42
(b)	Foods in Scenario 2a with the addition of:	
	NPSC-compliant RTEBC	8.33
(c)	Foods in Scenario 2b with the addition of:	
	Bread (plain and sweet bread, rolls and buns, damper, bagels, focaccia, english muffins, and flatbread)	1.7

*NPSC* Nutrient Profiling Scoring Criterion, *RTEBC* ready-to-eat breakfast cereals.

### Vitamin D food composition data

The analytical food composition data are described in detail elsewhere and briefly described here [30, 31]. Retail food and game products were procured across three states: Western Australia, Victoria and New South Wales. The samples of those food products were analysed using a validated liquid chromatography with triple quadrupole mass spectrometry method at the National Measurement Institute of Australia, a laboratory accredited by the National Association of Testing Authorities for measuring vitamin D in food (ISO17025:2017).

The analytical vitamin D concentrations were mapped to the 5740 food entries in the 2011–2013 AUSNUT database, following methods used for the Australian Total Diet Studies [32, 33]. This mapping involved assigning an analytical vitamin D concentration to each AUSNUT food entry [33]. Vitamin D concentrations were mapped either to single food items (e.g., milk) or to recipe foods (containing single food items as ingredients, e.g., custard) [32]. A conversion factor was used to adjust the analytical vitamin D values for foods that may undergo food manufacturing or preparation processes, such as the reconstitution of infant formula [32].

For each scenario, we created a modified vitamin D food composition dataset by adjusting vitamin D concentrations in the AUSNUT food database, which we mapped from the analytical food composition data [30, 31].

### Quantifying vitamin D intake for baseline and fortification scenarios

We modelled absolute vitamin D intake using day one of the 24-hour food recall data from the 2012–2013 NATSINPAS and the AUSNUT food database which included the analytical vitamin D concentrations. We used day one of the food recall data as day two had a low response rate (18.8%, *n* = 771) and was only conducted among those living non-remotely. Baseline vitamin D intake was quantified using the amount of individual food consumed by each respondent (g/day) multiplied by the vitamin D content (μg/g) of the food. Similarly, for each food fortification scenario, vitamin D intake was quantified by multiplying the amount of food consumed by each respondent (g/day) by the vitamin D content (μg/g) of the food in the modified food composition dataset for that scenario. For recipe foods consumed, a disaggregated 2011–2013 AUSNUT Food Recipe File [34] was used to identify single food items within a recipe, and the vitamin D concentrations for the recipe were adjusted according to the proportion of the vitamin D-containing ingredient/s within the recipe.

Given that the absolute vitamin D intake data were non-parametric, we extracted the 25th, 50th (median) and 75th percentile by sex and age groups for each of the four scenarios. A person-weighting variable developed by the ABS for NATSINPAS was applied to ensure that the results are representative of the total Aboriginal and Torres Strait Islander population [22].

The differences across the four scenarios were tested using a design-based Wald F-test, which adjusts for survey weights. Pairwise comparisons were conducted using survey-weighted mean differences. We compared the survey-weighted means, as the central limit theorem indicates that in large samples, the sampling distribution of the mean is approximately normal regardless of the raw data distribution [35]. Statistical significance was defined as *p* < 0.05. Statistical analysis was conducted using Stata version 17 (StataCorp) [36].

## Results

In the total group, median vitamin D intake increased from baseline by 3.6 μg/day, 3.2 μg/day, 4.2 μg/day, and 5.7 μg/day under Scenarios 1, 2a, 2b, and 2c, respectively (Table 2). The highest median (interquartile range) vitamin D intake was observed under Scenario 2c at 7.7 (7.5) μg/day, which included the greatest range of foods.

**Table 2:** Vitamin D intakes of baseline and food fortification scenarios among Aboriginal and Torres Strait Islander peoples aged ≥ 2 years.

Age group		Baseline^a^	Scenario 1^a,b^	Scenario 2a^a,b^	Scenario 2b^a,b^	Scenario 2c^a,b^
(years)	n	Median (25^th^, 75^th^ percentile) µg/day				
Total						
All ages	4109	2.0 (1.1, 3.6)	5.6 (3.1, 9.0)	5.2 (2.9, 9.0)	6.2 (3.4, 10.0)	7.7 (4.4, 11.9)
2–3	240	1.2 (0.8, 2.1)	7.6 (4.0, 10.6)	6.3 (3.3, 11.9)	8.3 (4.4, 11.9)	9.6 (5.5, 13.7)
4–8	515	1.8 (1.1, 3.0)	5.7 (3.4, 8.6)	4.9 (2.9, 8.2)	6.5 (3.8, 9.3)	8.0 (5.1, 11.2)
9–13	409	2.3 (1.3, 4.0)	6.3 (3.5, 9.0)	5.6 (3.5, 9.4)	7.1 (3.8, 10.3)	8.5 (5.2, 12.4)
14–18	332	1.9 (0.9, 3.8)	4.9 (2.3, 9.3)	5.2 (2.3, 8.2)	5.8 (2.5, 10.3)	6.8 (3.2, 12.2)
19–30	722	2.2 (1.2, 4.0)	5.2 (2.7, 9.0)	5.4 (2.8, 9.4)	5.9 (3.0, 9.9)	7.1 (4.0, 11.7)
31–50	1098	2.2 (1.3, 3.8)	5.1 (2.7, 8.8)	5.1 (2.8, 8.9)	5.8 (3.1, 9.4)	7.4 (4.0, 11.0)
51–74	722	2.0 (1.1, 3.4)	5.8 (3.3, 9.0)	5.4 (3.1, 9.0)	6.4 (3.6, 10.0)	8.1 (4.7, 11.6)
≥ 75	71	1.0 (0.8, 2.4)	4.3 (2.8, 7.5)	3.7 (2.4, 6.0)	4.6 (3.1, 8.0)	6.0 (4.1, 9.3)
**Male**						
All ages	1814	2.2 (1.3, 4.1)	6.3 (3.4, 10.1)	5.9 (3.3, 9.6)	6.9 (3.7, 11.1)	8.4 (4.9, 12.9)
2–3	125	1.3 (1.0, 1.8)	7.6 (4.4, 10.7)	6.5 (3.4, 12.7)	8.5 (4.9, 12.0)	10.2 (5.9, 13.7)
4–8	259	1.8 (1.1, 3.6)	5.7 (3.3, 9.5)	4.7 (2.8, 8.4)	6.0 (3.6, 10.3)	8.2 (4.8, 11.4)
9–13	207	2.5 (1.7, 4.2)	7.3 (3.9, 10.1)	6.5 (3.7, 10.1)	8.2 (4.2, 11.2)	9.8 (5.5, 13.1)
14–18	164	2.4 (1.2, 4.0)	6.8 (3.6, 11.0)	6.3 (3.6, 9.9)	7.7 (4.0, 11.8)	9.6 (5.5, 13.6)
19–30	299	2.4 (1.4, 4.8)	5.9 (3.4, 10.1)	6.2 (3.4, 10.7)	6.4 (3.7, 11.5)	8.2 (4.4, 13.8)
31–50	427	2.5 (1.5, 4.4)	5.8 (3.1, 9.7)	5.8 (3.4, 9.4)	6.5 (3.5, 10.5)	8.1 (4.7, 13.1)
51–74	308	1.9 (1.1, 3.5)	6.1 (3.2, 9.0)	5.1 (3.1, 9.1)	6.4 (3.5, 10.0)	8.1 (4.4, 12.1)
≥ 75	25	1.6 (0.9, 4.0)	6.5 (4.3, 11.1)	5.7 (3.1, 9.3)	7.4 (4.5, 12.8)	8.5 (5.8, 13.7)
**Female**						
All ages	2295	1.9 (1.0, 3.2)	5.1 (2.7, 8.0)	4.9 (2.6, 8.1)	5.6 (3.0, 8.9)	7.0 (4.1, 10.4)
2–3	115	1.1 (0.6, 2.4)	7.3 (3.8, 10.1)	5.9 (2.8, 11.9)	8.1 (4.0, 11.9)	9.3 (4.5, 13.7)
4–8	256	1.8 (1.0, 2.8)	5.9 (3.6, 8.2)	5.6 (3.2, 8.2)	6.6 (3.9, 8.8)	7.9 (5.6, 10.5)
9–13	202	2.2 (1.2, 3.4)	5.5 (3.0, 8.3)	5.2 (2.7, 8.2)	6.0 (3.5, 9.4)	8.2 (5.0, 11.1)
14–18	168	1.4 (0.7, 2.9)	3.8 (1.7, 6.2)	4.3 (1.9, 6.6)	4.3 (1.9, 6.9)	5.3 (2.3, 8.1)
19–30	423	2.0 (1.0, 3.4)	4.3 (2.3, 7.7)	4.4 (2.5, 8.2)	4.7 (2.5, 8.4)	6.2 (3.5, 9.9)
31–50	671	1.9 (1.0, 3.3)	4.6 (2.6, 7.5)	4.7 (2.6, 7.8)	5.2 (2.8, 8.5)	6.6 (3.7, 9.9)
51–74	414	2.0 (1.1, 3.4)	5.7 (3.6, 9.3)	5.9 (3.2, 8.9)	6.4 (3.9, 10.3)	8.1 (4.8, 11.5)
≥ 75	46	1.0 (0.8, 2.3)	2.9 (2.8, 7.2)	3.1 (2.2, 4.4)	3.3 (3.1, 7.7)	4.2 (4.1, 7.7)

^a^Weighted to the benchmark of Aboriginal and Torres Strait Islander estimated resident population living in private dwellings of Australia at 30 June 2011, based on the 2011 Census of Population and Housing, with survey weight provided by the Australian Bureau of Statistics.

^b^The modelling for Scenario 1 included the maximum vitamin D concentrations permitted for fortification in Australia: i) dairy products and alternatives, ii) butter/margarine/edible oil spreads, iii) formulated beverages, iv) selected ready-to-eat breakfast cereals. The modelling for Scenarios 2a-c included some vitamin D concentrations higher than permitted in Australia; Scenario 2c included bread, which is not currently permitted for vitamin D fortification in Australia. Scenario 2a: i) dairy products and alternatives, ii) butter/margarine/edible oil spreads, iii) formulated beverages. Scenario 2b: as per Scenario 2a plus selected ready-to-eat breakfast cereals. Scenario 2c: as per Scenario 2b plus bread.

Among children aged 2–18 years, those aged 2–3 years had the greatest median increase in vitamin D intake across all the scenarios, and the trend was consistent across both sex groups. In Scenario 2c, there was nearly an eight-fold increase in median vitamin D intake among those aged 2–3 years.

Among adults aged ≥19 years, the greatest increase in vitamin D intake across all scenarios was observed among those aged 51–74 years. Older female adults aged ≥75 years had the lowest median baseline vitamin D intake and the smallest increase in median vitamin D intake across all scenarios.

Across all the modelled scenarios, none of the participants had a vitamin D intake above the Australian upper level of intake of 80 μg/day [37].

We found significant statistical differences across all four scenarios (adjusted Wald F (3,16433) = 46.61, *p* < 0.001). Pairwise comparison showed a clear increase across the scenarios (Table 3). There were no significant differences between Scenarios 2a and 1 (difference in marginal means: −0.11 μg/day, 95% confidence interval (CI): −0.48–0.26, *p* = 0.559). The greatest statistically significant difference was observed between Scenarios 2c and 2a (difference in marginal means: 2.21 μg/day, 95% CI: 1.81–2.62, *p* < 0.001). All other pairs were significantly different (*p* < 0.001).

**Table 3:** Pairwise comparisons of marginal means across all food fortification scenarios^a,b^.

Scenarios	Difference in marginal means, µg/day^c^	95% confidence interval	p-value
2a & 1	−0.11	(−0.48, 0.26)	0.559
2b & 1	0.72	(0.33, 1.11)	< 0.001
2b & 2a	0.83	(0.44, 1.22)	< 0.001
2c & 1	2.10	(1.70, 2.50)	< 0.001
2c & 2a	2.21	(1.81, 2.62)	< 0.001
2c & 2b	1.38	(0.96, 1.80)	< 0.001

^a^Weighted to the benchmark of Aboriginal and Torres Strait Islander estimated resident population living in private dwellings of Australia at 30 June 2011, based on the 2011 Census of Population and Housing, with survey weight provided by the Australian Bureau of Statistics.

^b^The modelling for Scenario 1 included the maximum vitamin D concentrations permitted for fortification in Australia: i) dairy products and alternatives, ii) butter/margarine/edible oil spreads, iii) formulated beverages, iv) selected ready-to-eat breakfast cereals. The modelling for Scenarios 2a–c included some vitamin D concentrations higher than permitted in Australia; Scenario 2c included bread, which is not currently permitted for vitamin D fortification in Australia. Scenario 2a: i) dairy products and alternatives, ii) butter/margarine/edible oil spreads, iii) formulated beverages. Scenario 2b: as per Scenario 2a plus selected ready-to-eat breakfast cereals. Scenario 2c: as per Scenario 2b plus bread.

^c^Difference in the predicted population marginal means between two food fortification scenarios.

## Discussion

Vitamin D fortification of a range of staple foods among Aboriginal and Torres Strait Islander peoples could increase median vitamin D intakes by ~3–6 µg/day from a baseline of 2 µg/day. Scenario 2c had the highest median vitamin D intake at 7.7 µg/day; it included the broadest range of foods and had higher vitamin D concentrations for fluid milk and alternatives, and butter, margarine, and edible oil spreads. There was no statistically significant difference between Scenario 1 and 2a; however, the slight decrease in vitamin D intake between these two scenarios might be attributed to the removal of NPSC-compliant RTEBC in Scenario 2a. These findings highlight the importance of including a wide range of food vehicles to accommodate various food preferences. Excessive vitamin D intake may result in hypercalcemia and hypercalciuria [38]; however, none of the participants had a vitamin D intake that exceeded the Australian upper level of intake under any modelled scenarios.

Based on our previous dose-response analyses [39], a median increase of ~3 μg/day from food fortification may translate to an increase in mean serum 25(OH)D concentration of 10.1 (95% CI: 4.1–16.0) nmol/L in children and 6.8 (95% CI: 5.2–8.3) nmol/L in adults. If all foods permitted for fortification in Australia were fortified with their maximum permitted vitamin D concentration, the mean serum 25(OH)D concentration may increase between 10.1–16.7 nmol/L for children and 6.8–8.9 nmol/L for adults.

Our findings suggest that food fortification could be a suitable public health strategy to increase vitamin D intake and ultimately improve vitamin D status among the Aboriginal and Torres Strait Islander population. Elsewhere, food fortification practices differ by country depending on their legislation [40]. For example, in Finland, vitamin D fortification of fat spreads and fluid milk products is voluntary but was widely implemented by most manufacturers, and the prevalence of serum 25(OH)D concentration <50 nmol/L decreased from 56% in 2000 to 9% in 2011 [41]. In Canada, vitamin D fortification of cow’s milk and margarine is mandatory, while goat’s milk, plant-based beverages, yoghurt, and kefir may be fortified voluntarily [42–44]. In the United States, the vitamin D fortification of dairy products, fruit juices, and breakfast cereals is largely voluntary [45]. Yet, milk has been shown to be the greatest contributor to vitamin D intake because it is widely fortified [46]. The mandated vitamin D concentration for edible oil spreads in Australia is lower than that of margarine in Canada (26 µg/100 g) and fat spreads in Finland (20 µg/100 g) [41, 47]. For milk, the fortification level in Australia (0.8 µg/100 ml) is also lower than in Canada and the United States (approximately 2 µg/100 ml) [13, 47, 48]. However, increasing the vitamin D concentration allowed for fortification and expanding the range of foods permitted for vitamin D fortification and would require a change to the Australia New Zealand Food Standards Code.

Maintaining adequate vitamin D status may reduce the risk of falls and fractures [3]; serum 25(OH)D concentration <50 nmol/L may account for over 9000 hospitalisations annually for falls and hip fractures among older adults aged ≥65 years [49]. Falls are common among Aboriginal and Torres Strait Islander peoples, with ~8840 hospitalisations from falls in 2023–2024 [50]. Despite the inclusion of a wide range of foods in Scenario 2c, the median vitamin D intake among older female adults aged ≥75 years remained low at 4.2 µg/day, and low vitamin D status in this age group is associated with a greater risk of fractures [51]. International evidence suggests that food fortification could be cost-effective [52, 53]; for example, vitamin D and calcium food fortification in Germany could save approximately 315 million Euros annually by preventing osteoporotic fractures in older female adults [53]. Vitamin D food fortification could have similar cost-saving benefits in Australia.

Mandatory folate fortification has proven to be successful, reducing neural tube defects by approximately 74% among babies of Aboriginal and Torres Strait Islander mothers in New South Wales, Queensland, Western Australia, South Australia, and the Northern Territory from the baseline period (2006–2008) to after implementation (2009–2011) [54]. Successful implementation of food fortification as a public health strategy requires careful consideration of health equity across the Aboriginal and Torres Strait Islander population. This includes addressing the ongoing challenge of food insecurity, especially among those living remotely, as food supplies are often disrupted due to transportation issues caused by weather or poor road infrastructure [55–57]. Fortified foods must therefore be affordable, shelf-stable, and culturally appropriate. Our modelling included shelf-stable foods such as RTEBC and dried milk. In addition to these considerations, the selection of food vehicles should also respect the food sovereignty of Aboriginal and Torres Strait Islander peoples. As any food fortification policy would be implemented nationally, consultations with Aboriginal and Torres Strait Islander Elders and knowledge holders are essential prior to implementation. Their guidance can help to ensure that proposed measures are feasible and culturally appropriate, and that they complement, rather than replace, their traditional food practices such as hunting and gathering.

Other complementary food-based strategies, such as routine biofortification of foods (e.g., through exposure of foods that contain a vitamin D precursor, such as mushrooms, to ultraviolet light, or vitamin D supplementation of animal feed [58]), or supplementation, could also be explored. Supplementation could be a suitable complementary strategy and has been shown to be useful in achieving adequate vitamin D intakes among multi-ethnic population which include the Sami people in northern Norway [59]. However, as overall vitamin and mineral supplement use among Aboriginal and Torres Strait Islander peoples was reported to be low (~18%) in the 2023–2024 NATSINPAS [60], culturally appropriate messaging would be essential to increase the awareness of vitamin D, its health benefits, and the safe use of supplementation. There are currently no recommendations for routine vitamin D supplement use among the general Australian population in Australia, except for those in high-risk groups (e.g., osteoporosis) [61].

While our modelling demonstrated that food fortification could increase vitamin D intake, obtaining vitamin D through traditional foods should still be prioritised. Traditional food and food systems are an integral part of the culture of Aboriginal and Torres Strait Islander peoples and how they connect with Country [62]. Before colonisation, Aboriginal and Torres Strait Islander peoples consumed nutritious traditional foods, including a wide variety of native plant foods such as fruits, root vegetables, and nuts, as well as animal foods such as game meats and fish, protecting them from many current nutrition-related health conditions [1]. The scientific literature regarding traditional foods and the nutritional composition of traditional foods is still limited. Yarning is a cultural form of communication that involves storytelling and conversations, commonly used by Aboriginal and Torres Strait Islander peoples to build relationships and share knowledge [63]. Yarning with Elders and Aboriginal and Torres Strait Islander peoples who are knowledge holders within the community is important to learn more about their traditional food practices. The findings will be beneficial in guiding food-based strategies to improve vitamin D intake within this population group.

Similar modelling has recently been conducted among the general Australian population [64]. The results showed that vitamin D intake could increase by ~7 μg/day from baseline, and ~40% of the population could meet the estimated average requirement of 10 μg/day if fortification included a broader range of foods, such as bread, and higher-than-permitted vitamin D concentrations were applied to some foods. Given that low vitamin D status and low intake ( < 3.5 μg/day) are also prevalent across the general Australian population [33]; food fortification is likely to benefit the wider Australian population.

A strength of our study was the use of analytical vitamin D food composition data containing four D vitamers to quantify potential vitamin D intakes. Some limitations should be considered when interpreting the results. Single-day food consumption data were used to quantify median vitamin D intakes in the modelling scenarios, as day two of the 24-hour recall interview was conducted in non-remote areas only and had limited responses (*n* = 771). To capture any potential seasonality in vitamin D intake, the NATSINPAS was conducted across 12 months [25]. However, single-day food recall does not capture any within- and between-person variation, or intake from episodically consumed foods, such as fish [65, 66].

The Automated Multiple-Pass Method is a structured interview approach designed to improve the accuracy of 24-hour food recalls; however, under-reporting in the NATSINPAS is likely, as this method relies on memory and may result in unintentional inaccuracies in reporting [25, 65]. We did not include supplement use in our models as only ~12% of participants reported using any supplement use, indicating that it would not significantly impact our models [25].

Although the food consumption data were collected in 2012–2013, they are the most recent data available for modelling. Further, there has been no major change to policy relating to vitamin D fortification, or in the availability of vitamin D-enhanced foods [64].

Our findings demonstrate that fortifying a range of commonly consumed staple foods with vitamin D has the potential to increase vitamin D intake among Aboriginal and Torres Strait Islander peoples. For maximum benefit, a revision of the Australia New Zealand Food Standards Code would be required to permit higher levels of vitamin D concentration for fortification in fluid milk and alternatives, and butter, margarine, and edible oil spreads, and to expand the list of foods permitted for vitamin D fortification to include bread.

## Supplementary information

Supplementary file

## Data Availability

Data described in the manuscript were sourced from publicly available datasets found here: 2012–2013 National Aboriginal and Torres Strait Islander Nutrition and Physical Activity Survey https://www.abs.gov.au/statistics/microdata-tablebuilder/microdatadownload, AUSNUT 2011–2013 https://www.foodstandards.gov.au/science-data/monitoringnutrients/afcd/australian-food-composition-database-download-excel-files#nutrient and vitamin D food composition data https://www.foodstandards.gov.au/science-data/monitoringnutrients/afcd/Data-provided-by-food-companies-and-organisations.
