# Supplementary material for: Modelling vitamin D food fortification among Aboriginal and Torres Strait Islander peoples in Australia
Source: Eur J Clin Nutr. 2026 Apr 9;80(7):686–93. doi: 10.1038/s41430-026-01737-y (PMC13341319; doi:10.1038/s41430-026-01737-y)
Supplement: Supplementary file 1 — Supplementary file [file 41430_2026_1737_MOESM1_ESM.docx]

**Supplementary Table 1.** Proportion of food group consumption and mean daily energy intake from food groups among Aboriginal and Torres Strait Islander peoples aged ≥ 2 years^a,b^

| **Food groups^c^** | **Persons (%)** | **mean kJ/day** |
| --- | --- | --- |
| Dairy milk (includes fluid and dried milk) | 69.3 | 385.3 |
| Dairy milk substitutes, unflavoured (includes alternatives derived from legumes, cereals, or nuts) | 0.8 | 2.6^d^ |
| Fats and oils (includes butters, dairy blends, margarine and table spreads, and oils) | 56.2 | 177.5 |
| Cheese | 26.4 | 145.5 |
| Yoghurt | 7.3 | 44.7 |
| Frozen milk products (includes dairy desserts) | 12.0 | 121.0 |
| Waters, municipal and bottled, unflavoured | 81.9 | 0.2^e^ |
| RTEBC | 33.8 | 238.1 |
| Bread | 70.3 | 787.5 |

kJ, kilojoules; RTEBC, ready-to-eat breakfast cereal

^a^ Weighted to the benchmark of Aboriginal and Torres Strait Islander estimated resident population living in private dwellings of Australia at 30 June 2011, based on the 2011 Census of Population and Housing^1^

^b^ Based on data extracted from the 2012-2013 National Aboriginal and Torres Strait Islander Nutrition and Physical Activity Survey nutrition results^2^

^c^ Based on the 2011-13 AUStralian Food and NUTrient Database classification^3^

^d^ Estimate provided by the Australian Bureau of Statistics has a relative standard error of 25% to 50%, interpret with caution^2^

^e^ Estimate provided by the Australian Bureau of Statistics has a relative standard error of >50%, considered unreliable^2^

**References**

1. Australian Bureau of Statistics. Australian Aboriginal and Torres Strait Islander Health Survey: users' guide, 2012-13. 2013. https://www.abs.gov.au/ausstats/abs@.nsf/PrimaryMainFeatures/4727.0.55.002?OpenDocument.
2. Australian Bureau of Statistics. Australian Aboriginal and Torres Strait Islander Health Survey: nutrition results - food and nutrients, 2012-13. 2015. https://www.abs.gov.au/ausstats/abs@.nsf/Lookup/by%20Subject/4727.0.55.005~2012-13~Main%20Features~Key%20Findings~1.
3. Food Standards Australia New Zealand. AUSNUT 2011-2013. 2016. https://www.foodstandards.gov.au/science-data/monitoringnutrients/ausnut/ausnutdatafiles#food-dietary
